# Irritable bowel syndrome-specific health-related quality of life instrument: development and psychometric evaluation

**DOI:** 10.1186/s12955-016-0423-9

**Published:** 2016-02-17

**Authors:** Eun-Hyun Lee, Oran Kwon, Ki Baik Hahm, WonHee Kim, Jin Il Kim, Dae Young Cheung, Yoon Jae Kim, Jung Ho Kim, Jong-Jae Park, Moon Kyung Joo

**Affiliations:** Graduate School of Public Health, Ajou University, 164 Worldcup-ro, Yeongtong-gu, Suwon-si, Gyeonggi-do 443-380 Republic of Korea; Department of Food & Nutritional Sciences, Ewha Womans University, Seoul, Republic of Korea; Digestive Disease Center, CHA Bundang Medical Center, CHA University, Seongnam, Republic of Korea; Department of Internal Medicine, The Catholic University of Korea, Yeouido St. Mary’s Hospital, Seoul, Republic of Korea; Department of Internal Medicine, Gachon University Gil Medical Center, Incheon, Republic of Korea; Division of Gastroenterology, Department of Internal Medicine, Korea University Guro Hospital, Seoul, Republic of Korea

**Keywords:** Irritable bowel syndrome, Health-related quality of life, Reliability, Validity

## Abstract

**Background:**

Health-related quality of life (HRQOL) is an important outcome indicator for chronic disease, and particularly in the absence of biological markers for illness, such as with irritable bowel syndrome (IBS). The aims of this study were to develop and evaluate a new IBS-specific HRQOL instrument (IBS-HR-QOL).

**Methods:**

This methodological study comprised three steps: conceptualization of the IBS-HR-QOL, item extraction and establishment of content validity, and psychometric evaluation of the instrument with 267 IBS patients recruited from four university hospitals.

**Results:**

The content validity of the developed IBS-HR-QOL was assessed by 11 experts. Exploratory and confirmatory factor analyses yielded four factors. The criterion and convergent validities of the IBS-HR-QOL were demonstrated using the Short Form-36 and the Hospital Anxiety and Depression Scale, respectively. Known-groups validity was demonstrated using a symptom-severity scale. The internal consistency reliability and test-retest reliability were satisfactory, with a Cronbach’s alpha and intraclass correlation coefficient of 0.93 and 0.88, respectively.

**Conclusions:**

The IBS-HR-QOL comprises a total of 16 items. The IBS-HR-QOL demonstrated good psychometric properties. This instrument is easily comprehensible and short, rendering it feasible for use in clinical practice and research.

**Electronic supplementary material:**

The online version of this article (doi:10.1186/s12955-016-0423-9) contains supplementary material, which is available to authorized users.

## Background

Irritable bowel syndrome (IBS) is a functional gastrointestinal disorder that is characterized by abdominal pain or discomfort associated with altered bowel habits, with an uncertain organic cause [1]. It is a common condition with a global prevalence of 11.2 % [2], conferring a substantial health-related economic burden upon society, and significantly impairing the health-related quality of life (HRQOL) at the individual level [3].

HRQOL is considered an important outcome indicator for chronic disease or illness, since the traditional indicators that are used in clinics (e.g., physical, physiological, or biochemical indicators) cannot fully assess the impact of disease or its treatment on patients [4]. Measurement of HRQOL to evaluate and follow-up disease is of particular importance in the case of illnesses for which there are currently no biological markers, such as IBS. In the clinical management of IBS patients, an HRQOL assessment may help health professionals to check responses to treatment, facilitate communication with the patients or their families, and deliver optimal care to the patients [5]. Therefore, the American College of Gastroenterology has recommended the routine assessment of HRQOL in IBS patients [1]. The gastroenterology division of the United States Food and Drug Administration has recommended evaluation of HRQOL in clinical trials of IBS treatments [6].

There exist generic and disease-specific HRQOL instruments. Generic instruments can be used to assess healthy populations or for comparisons with other disease populations, while disease-specific instruments are used to assess patients with a particular disease, since they are more sensitive to the patients’ condition or concerns [7]. Several IBS-specific HRQOL instruments have been developed over the last 17 years: Irritable Bowel Syndrome Quality of Life (IBSQOL) [8], Irritable Bowel Syndrome-Quality of Life (IBS-QOL) [9], Irritable Bowel Syndrome Health Related Quality of Life (IBS-HRQOL) [10], IBS-36 [11], and IBS Impact Scale (IBS-IS) [12]. However, these existing instruments have some methodological limitations. For example, all except the IBSQOL [8] were developed and tested using samples that were too small. The structural validity of patient-reported outcome instruments is usually evaluated using exploratory factor analysis (EFA) and confirmatory factor analysis (CFA); the former is used to identify underlying factors or item reduction, while the latter is used to assess the extent to which the factors proposed by the EFA fit the data [13]. Although the underlying factors of the IBS-QOL [9], IBS-IS [12], and IBS-HRQOL [10] were assessed using EFA, the results were unclear and/or the underlying factors were not subsequently evaluated using CFA. Hence, these limitations may threaten the findings of clinical trials or interventions in which these instruments are used as an outcome parameter. The aims of this study were to develop a new IBS-specific HRQOL instrument (called IBS-HR-QOL), and to test the psychometric properties of the instrument in patients with IBS.

## Methods

### Step 1: conceptualization

The fundamental consideration in the development of an instrument is defining the concept that is to be measured. Although there is no universally accepted definition of HRQOL, the following attributes have been widely agreed upon: a) HRQOL is subjective, and hence it depends upon the individual’s perception of the impact of his/her disease and its treatment on various aspects of his/her health-related life, and b) HRQOL comprises multidimensional constructs [14]. HRQOL has been defined as “the assessment of the impact of disease and treatment across the physical, psychological, social, and somatic domains of functioning and well-being” [15]. Combining attributes and implication, the concept of HRQOL being measured in the present study was defined as the individual’s subjective perception of the effects of IBS on various aspects of his/her the health-related life, such as somatic, dietary, emotional, and social aspects.

### Step 2: item extraction and content validity

A pool of 48 attributes was derived from literature reviews on qualitative and quantitative studies, and from discussions with 4 clinicians. Each attribute was formulated into an item. A 5-point Likert scale was used as a response format for the items, ranging from 0 (“not at all”) to 4 (“very much”); this is the most frequently used response format in survey questionnaires [16]. The content validity of the preliminary questionnaire was evaluated by a panel of 11 experts (7 gastroenterologists and 4 experts on concept analysis). A content validity ratio (CVR) was calculated for each item. A minimum CVR of 0.59 was considered as a threshold value at *p* < 0.05 [17].

### Step 3: psychometric evaluation

#### Sample and data collection procedures

A convenience sample of 267 patients with IBS was recruited from outpatient clinics at 4 university hospitals in South Korea (Table 1). The inclusion criteria were being aged at least 20 years, articulate in Korean, diagnosed with IBS by gastroenterologists based on the Rome III criteria [18], and an absence of abnormal results on a colonoscopic examination.

**Table 1:** Characteristics of the patients

Variable	*n* (%)
Gender	
Male	110 (41.2)
Female	157 (58.8)
Age in years (Mean ± SD = 46.69 ± 14.57 years)	
20–29	36 (13.5)
30–39	56 (21.0)
40–49	58 (21.7)
50–59	64 (23.9)
60–69	35 (13.2)
≥ 70	18 (6.7)
Marital status	
Married/living together	191 (71.6)
Divorced/widow(er)	17 (6.4)
Unmarried	54 (20.2)
Other	2 (0.7)
Missing value	3 (1.1)
Education	
Elementary school	14 (5.3)
Middle school	22 (8.2)
High school	71 (26.6)
College and above	157 (58.8)
Other	1 (0.4)
Missing value	2 (0.7)
IBS subtype	
IBS-C	41 (15.3)
IBS-D	143 (53.6)
IBS-M	47 (17.6)
IBS-U	36 (13.5)

IBS-C, constipation-predominant IBS; IBS-D, diarrhea-predominant IBS; IBS-M, mixed-symptom IBS; IBS-U, IBS subtype unknown

Potential patients who met the inclusion criteria were recruited by research assistants at outpatient clinics. The research assistants met those potential patients who agreed to participate in this study in a small private room, and informed them about the purpose of the study, the confidentiality of their data, and their right to withdraw from the study at any time. They were then asked to sign on a written consent form. Thereafter the patients were provided with a package of questionnaires and asked to complete them in the private room.

The test–retest reliability of an instrument can be assessed by applying it at least twice to the same individuals with an interval of 1–2 weeks [9, 11]. Therefore, some of the participants in this study were required to complete the IBS-HR-QOL instrument twice, with a 1-week interval. In total, 59 of the patients agreed to provide repeat responses to the IBS-HR-QOL questionnaire. These patients were given an envelope with a returning address and a stamp, in which an uncompleted IBS-HR-QOL questionnaire was enclosed, for the assessment of test–retest reliability. They were asked to take the envelope home, complete the IBS-HR-QOL 1 week later, and then post the return envelope to the researchers.

### Ethical considerations

This study was approved by the institutional review boards of the hospitals at which the participants were recruited (IRB reference numbers BD2013-094, SIRB-00200-2-002, GBIRB2013-251, and KUGH13183-001).

### Measures

#### Short Form-36 (SF-36)

The SF-36 was used to test the criterion validity of the IBS-HR-QOL. The SF-36 measures generic HRQOL [19], and comprises eight subscales (physical functioning, role physical, bodily pain, role emotional, vitality, mental health, social functioning, and general health), with higher scores indicating a better HRQOL. The reliability and validity of the Korean version of the SF-36 have been demonstrated in 2,511 Koreans [20]. In the present study it was hypothesized that the IBS-HR-QOL would be moderately positively correlated with the SF-36, since a disease-specific HRQOL is generally known to be moderately correlated with generic HRQOL instruments [21].

#### IBS Symptom-Severity Scale (IBS-SSS)

The IBS-SSS, which measures lower-gastrointestinal symptom severity, was used to assess known-groups validity [22]. The IBS-SSS consists of five items, and the total score ranges from 0 to 500. Participants with scores of <175, 175–300, and >300 were classified as mild, moderate, and severe groups, respectively. In this study, it was hypothesized that the IBS-HR-QOL score would be higher for a more severe IBS-SSS classification [9, 23].

#### Hospital Anxiety and Depression Scale (HADS)

The HADS, which measures anxiety and depression in people with illness, was applied in this study [24]. HADS scores are summed, with higher scores indicating a higher level of anxiety and depression. The HADS has previously been demonstrated to have satisfactory reliability and validity in a Korean population [25]. A prior hypothesis for convergent validity in the present study was that there would be a moderate correlation between the HADS and IBS-HR-QOL scores [12].

### Data analysis

The Statistical Package for the Social Sciences (version 20) and AMOS were used to analyze the data. A zero-order correlation matrix was computed to identify uncorrelated or redundant items. If the interitem correlation coefficient was <0.3 or >0.8, the item was eliminated [26].

Regarding structural validity, the required sample size was at least seven times the number of items, but with 100 individuals as an absolute minimum [27]. The sample size included in this study met this requirement. Bartlett’s test of sphericity and the Kaiser-Mayer-Olkin measure of sampling adequacy (KMO) were computed to justify undertaking a factor analysis. EFA was conducted to reduce the items and explore their underlying structure, using principal-components analysis with varimax rotation. Factors with an eigenvalue of >1 were extracted. The criterion for factor loadings was set at ≥0.60 [28].

The fit of the underlying structure to the observed data were confirmed through CFA using a maximum-likelihood method. The CFA model fit was assessed using multiple fit indices and their acceptable criteria: the normed *χ*^2^ [the ratio of *χ*^2^/*df* < 3], goodness-of-fit index (GFI > 0.9), standardized root mean square residual (SRMR < 0.05), root-mean-square error of approximation (RMSEA < 0.08), comparative fit index (CFI > 0.90), and normed fit index (NFI > 0.90) [29, 30].

As an ancillary test of the structural validity, multigroup CFA (MGCFA) was performed to determine whether or not the proposed factor model was invariant across the IBS-subtype groups. First, a configure model (Model 1) was used to assess whether or not the factor structure was similar across the groups. Factor-loading invariance (Model 2: by constraining factor loadings to be the same across groups), covariance invariance (Model 3: by constraining factor loadings and covariance), and error variance invariance (Model 4: by constraining factor loadings, covariance, and error variance) were then also used. The MGCFA assessment was achieved with RMSEA (<0.08) and *χ*^2^ difference (∆*χ*^2^). If the ∆*χ*^2^ of the sequential comparisons between the models was not significant, the invariance across groups was satisfied [29].

Criterion and convergent validities were examined using the SF-36 and HADS, respectively, with Pearson’s correlation. The known-groups validity of the IBS-HR-QOL according to the IBS-SSS group was tested using ANOVA or Welch’s test (the latter was used when the homogeneity of variances among three groups was violated). Cohen’s effect size was computed to assess the magnitude of known-groups validity [4, 31, 32].

Internal consistency reliability was tested using corrected item-total correlations and Cronbach’s alpha; a Cronbach’s alpha value of 0.70–0.95 was acceptable for internal consistency [27]. Test–retest reliability was examined using the intraclass correlation coefficient (ICC); the threshold ICC value for acceptable test–retest reliability was set at ≥0.70 [27].

## Results

### Content validity

Of the 48 items, 29 satisfied the threshold CVR value of >0.59. The remaining 18 items were deleted. The experts reported that three items were difficult to understand and that two items were ambiguous to answer. Those three items were thus modified so that they were clearer and more comprehensible.

### Missing values and correlation matrix

The score for each item ranged from 1.60 ± 1.28 to 3.53 ± 0.95. The rate of missing values for each item ranged from 0 to 1.5 %. The missing values were completed using expectation-maximization. In the 29 × 29 correlation matrix, one item (“sexual relationship”) was not significantly correlated with more than half of the other items, and was thus eliminated.

### Structural validity

Bartlett’s sphericity was significant (*χ*^2^ = 5424.05, *p* < 0.001). The KMO was superb, at 0.94, implying that a factor analysis could be used to identify factors [33]. The initial EFA extracted a four-factor solution, which accounted for 64.47 % of the total variance. Twelve items did not load onto any of the four factors at the criterion value. After excluding these 12 items, consecutive factor analysis again extracted a 4-factor solution, but the explained variance had increased to 72.93 %. All 16 items meaningfully loaded onto 1 of the 4 factors. No item loaded onto cross factors above the criterion value of 0.6 (Table 2). The four were named as “bowel function,” “emotions,” “concerns about social activity,” and “consideration of foods.”

**Table 2:** Factor loadings

No.	Abbreviated item	Factor 1	Factor 2	Factor 3	Factor 4	*h* ^2^
1	Abdominal bloating	**0.78**	0.22	0.10	0.14	0.69
2	Tenesmus	**0.74**	0.21	–0.00	0.12	0.61
3	Changed stool form	**0.68**	0.16	0.37	0.12	0.65
4	Changed stool frequency	**0.69**	0.03	0.39	0.15	0.65
5	Flatulence	**0.73**	0.22	0.16	0.22	0.66
6	Depression	0.24	**0.77**	0.12	0.27	0.73
7	Irritation	0.34	**0.69**	0.25	0.27	0.73
8	Angry	0.09	**0.81**	0.18	0.14	0.71
9	Nervousness	0.26	**0.77**	0.20	0.26	0.76
10	Finding a toilet	0.20	0.32	**0.77**	0.25	0.70
11	Holding back defecation	0.20	0.10	**0.89**	0.18	0.75
12	Unpredictability	0.21	0.26	**0.80**	0.21	0.84
13	Eating before going out	0.24	0.22	0.46	**0.63**	0.74
14	Consideration of foods	0.20	0.23	0.15	**0.80**	0.80
15	Favorite foods	0.16	0.21	0.09	**0.87**	0.86
16	Eating out	0.15	0.28	0.35	**0.72**	0.79

Bartlett’s sphericity: *χ* ^2^ = 2676.64, *p* < 0.001, KMO = 0.90

Factor 1, bowel function; Factor 2, emotions; Factor 3, concerns about social activity; Factor 4, consideration of foods; *h* ^2^, communality of the measured variables

Bold values: meaningful loadings

Original full version: see additional file 1

**Fig. 1:**
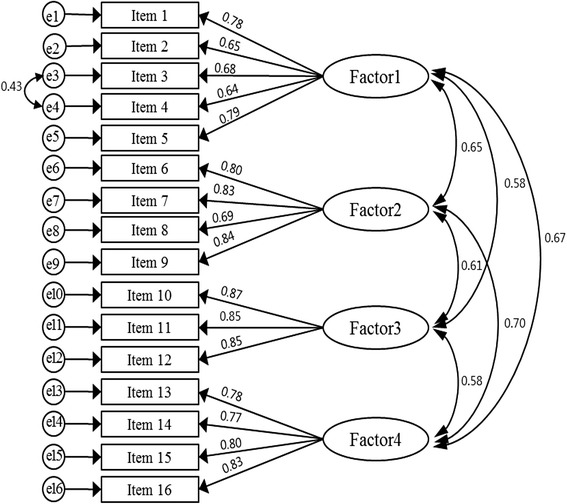
Confirmatory factor analysis for the IBS-HR-QOL. Factor 1, bowel function; Factor 2, emotions; Factor 3, concerns about social activity; Factor 4, consideration of foods; e, error term

CFA was computed for the fit of the extracted four-factor model to the data. The normed *χ*^2^ = 2.97, SRMR = 0.05, GFI = 0.88, RMSEA = 0.08 (90 % CI = 0.07–0.09), NFI = 0.89, IFI = 0.93, and CFI = 0.93. The GFI, RMSEA, and NFI were either not satisfied or did not reach the criterion cutoff. Therefore, explorative post-hoc inspection of model misspecification was carried out using modification indices (MIs). It was found that the error terms between items 3 and 4 exhibited a high MI (32.42). After fitting the item error covariance, *χ*^2^ decreased to 250.59 (∆*χ*^2^ = 40.76, *p* < 0.001), implying significant improvement of the model. The modified model fit indices were also improved and fitted the data well: normed *χ*^2^ = 2.97, SRMR = 0.05, GFI = 0.90, RMSEA = 0.07 (90 % CI = 0.06–0.08), NFI = 0.91, IFI = 0.94, and CFI = 0.94. The item loadings to the factors ranged from 0.64 to 0.87 (Fig. 1).

Regarding the ancillary analysis for structural validity, Table 3 presents the configural model of the four-factor model with one item-error covariance revealed *χ*^2^/*df =* 1.81 and RMSEA = 0.06, which indicated an acceptable fit across the IBS-subtype groups. The difference in *χ*^2^ between Models 1 and 2 was not statistically significant (∆*χ*^2^ = 38.57, ∆*df* = 36, *p* = 0.354), indicating that factor loadings were invariant across the groups. The *χ*^2^ differences between Models 2 and 3 (∆*χ*^2^ = 32.24, ∆*df* = 30, *p* = 0.357), and between Models 3 and 4 (∆*χ*^2^ = 58.95, ∆*df* = 51, *p* = 0.207) were also not significant. Therefore, the factor structure of the IBS-HR-QOL was invariant across the IBS-subtype groups.

**Table 3:** Tests for multigroup invariance across the IBS-subtype groups

	*χ* ^2^ (*df)*	*χ* ^2^/*df*	RMSEA (90 % CI)	Comparison	∆*χ* ^2^ (∆*df)*	*p*
Model 1^a^	704.92 (388)	1.81	0.06 (0.05–0.06)	-	-	-
Model 2^b^	743.48 (424)	1.75	0.05 (0.05–0.06)	Model 1 vs. Model 2	38.57 (36)	0.354
Model 3^c^	775.72 (454)	1.71	0.05 (0.04–0.06)	Model 2 vs. Model 3	32.24 (30)	0.357
Model 4^d^	834.67 (505)	1.65	0.05 (0.04–0.06)	Model 3 vs. Model 4	58.95 (51)	0.207

^a^Configural Model: No equality constraints imposed

^b^All factor loadings constrained equal

^c^All factor loadings and covariances among 4 factors constrained equal

^d^All factor loadings, covariances among 4 factors, and error variance constrained equal

∆*χ* ^2^= difference in *χ* ^2^ values between models

∆*df* = difference in number of degrees of freedom between models

### Criterion validity

As hypothesized, the IBS-HR-QOL was moderately correlated with the following four subscales of the SF-36: role physical (*r* = 0.41, *p* < 0.001), bodily pain (*r* = 0.41, *p* < 0.001), social functioning (*r* = 0.49, *p* < 0.001), and role emotional (*r* = 0.47, *p* < 0.001), and more weakly correlated with mental health (*r* = 0.36, *p* < 0.001), vitality (*r* = 0.29, *p* < 0.001), general health (*r* = 0.25, *p* < 0.001), and physical functioning (*r* = 0.25, *p* < 0.001).

### Known-groups validity

The IBS-HR-QOL scores for mild, moderate, and severe symptom severities were 2.97 ± 0.70, 2.22 ± 0.83, and 1.81 ± 0.94, respectively (Table 4). The mean differences overall were significant (*F* = 41.79, *p <* 0.001, *ƞ*^2^ = 0.24). A post-hoc inquiry revealed that the mean IBS-HR-QOL scores were higher in the mild symptom group than in the moderate and severe symptom groups at *p <* 0.001.

**Table 4:** Known-groups validity according to IBS severity classification

	Classification of IBS severity			*F*	Post-hoc test	Effect size (*η* ^2^)
	Mild	Moderate	Severe			
	(*n* = 101)	(*n* = 115)	(*n* = 45)			
	Mean ± SD	Mean ± SD	Mean ± SD			
Total	2.97 ± 0.70	2.22 ± 0.83	1.81 ± 0.90	41.79^a^*	Mild>Moderate, Severe^b^	0.24
F1	2.42 ± 0.90	1.57 ± 0.94	1.22 ± 0.97	34.49*	Mild>Moderate>Severe^c^	0.21
F2	3.11 ± 0.80	2.28 ± 1.00	1.71 ± 0.94	46.84^a^*	Mild>Moderate, Severe^b^	0.25
F3	3.14 ± 1.03	2.49 ± 1.27	2.15 ± 1.31	14.22^a^*	Mild>Moderate, Severe^b^	0.10
F4	3.20 ± 0.83	2.54 ± 1.13	2.17 ± 1.16	21.04^a^*	Mild>Moderate>Severe^b^	0.13

Factor 1, bowel function; Factor 2, emotions; Factor 3, concerns about social activity; Factor 4, consideration of foods

^a^Welch test; ^b^Games-Howell test; ^c^Scheffé’s test

*p < 0.001

### Convergent validity

As the predefined hypothesis, the IBS-HR-QOL score was correlated with the anxiety and depression subscales of the HADS, but the magnitudes of the correlations were only moderate (*r* = –0.43, *p <* 0.001) and small (*r* = –0.30, *p <* 0.001), respectively.

### Internal consistency reliability

The corrected item-total correlations for all items ranged from 0.50 to 0.70. Cronbach’s alpha value of the total IBS-HR-QOL was 0.93, which met the threshold criterion range of 0.70–0.95. Cronbach’s alpha of each subscale ranged from 0.85 to 0.90 (Table 5).

**Table 5:** Internal consistency reliability and test-retest reliability

	Cronbach’s alpha	ICC (95 % confidence interval)
Total IBS-HR-QOL	0.93	0.88 (0.79–0.86)*
Factor 1: Bowel function	0.85	0.81 (0.68–0.88)*
Factor 2: Emotions	0.87	0.82 (0.69–0.89)*
Factor 3: Concerns about social activity	0.90	0.88 (0.79–0.92)*
Factor 4: Consideration of foods	0.87	0.77 (0.61–0.86)*

*p < 0.001

### Test–retest reliability

The ICC of the total IBS-HR-QOL was 0.88 (confidence interval = 0.79–0.86, *p <* 0.001), while those of subscales 1 to 4 were 0.81 (confidence interval = 0.68–0.88, *p <* 0.001), 0.82 (confidence interval = 0.69–0.89, *p <* 0.001), 0.88 (confidence interval = 0.79–0.92, *p <* 0.001), and 0.77 (confidence interval = 0.79–0.92, *p <* 0.001), respectively (Table 5). Therefore, the temporal stability of the total and subscales was demonstrated.

## Discussion

The IBS-HR-QOL, which was developed and evaluated in this study, comprises a total of 16 items. This instrument consists of four subscales: bowel function, emotions, concerns about social activity, and consideration of foods. In this study, the four subscales were clustered by EFA and supported by CFA. To the best of our knowledge, this is the second study that has applied both EFA and CFA to test the structural validity of an IBS-specific HRQOL instrument. The first such study was conducted by Andrae et al. [34] with the IBS-QOL [9] in diarrhea-predominant IBS patients. And, the authors suggested that the IBS-QOL [9] was one dimensional in the diarrhea-predominant patients. However, the single dimension is inconsistent with the conceptual background of the original IBS-QOL instrument [9], and with the worldwide consensus of the multidimensionality of HRQOL [4]. To establish whether or not the underlying structure of the IBS-QOL in diarrhea-predominant IBS patients varied in comparison with that in other subtypes of IBS patients, it would be more appropriate to apply MGCFA [29]. From that perspective, MGCFA was used in the present study as an ancillary analytical tool, and demonstrated that the four-factor structural model of the IBS-HR-QOL was equivalent across the IBS-subtype groups. In other words, the IBS-HR-QOL may be used regardless of the patient’s IBS subtype. However, it should be noted that the present sample was too small to enable an unequivocal interpretation of the MGCFA findings. Thus, further study is required with sufficiently large samples to enable a robust MGCFA across the IBS subtypes.

Criterion validity is the degree to which the measurement instrument is an adequate reflection of a gold standard, and is satisfied if the correlation with the gold standard is at least 0.7 [27]. The SF-36 is generally accepted as a gold-standard measure of HRQOL in IBS research [35]. However, the SF-36 is a generic HRQOL instrument, and its likelihood of accurately measuring clinically important changes is lower than that of a disease-specific HRQOL instrument [7]. Even so, the SF-36 and disease-specific HRQOL instruments measure some common attributes. Therefore, the cutoff correlation value for the criterion validity between the SF-36 and IBS-specific instruments was considered to be ≥0.4 [11], which is lower than that generally used (>0.70). In the present study, the IBS-HR-QOL was correlated with four subscales of the SF-36 (role physical, social functioning, role emotional, and bodily pain) at >0.4. These findings are similar to those of validity testing of the IBS-36 [11] and IBS-QOL [9] relative to the SF-36.

Known-groups validity is associated with expected differences between subgroups of patients [36]. In the present study, the overall IBS-HR-QOL and all of its subscales satisfied the known-groups validity with the IBS-SSS classification. The effect sizes of the group differences for the overall IBS-HR-QOL were moderate based on Cohen’s criteria [37] for small (0.10), moderate (0.25), and large (0.40) effects. However, those of two subscales (“concerns about social activity” and “consideration of foods”) were small, requiring considerable interpretation.

Convergent validity refers to the extent to which a new scale is correlated with a well-established related measure according to a priori expectations [36]. The present study demonstrated that the IBS-HR-QOL satisfied the construct validity of a moderate correlation with the anxiety subscale of the HADS, as predicted by the study hypothesis. However, there was a weak correlation between IBS-HR-QOL and the depression subscale. This weaker correlation of the depression subscale compared with the anxiety subscale was also noted in the validity test of IBS-IS with HADS [12]. In line with this finding, it is recommended that in practice, anxiety rather than depression should be managed as one way of improving the HRQOL of IBS patients [38].

Internal consistency reliability refers to the extent to which items are homogeneous, and thus measure the same construct [27]. Existing HRQOL instruments for IBS patients presented a Cronbach’s alpha value that is either too high (>0.95), implying the redundancy of one or more items [34, 39], or too low (<0.70), inferring a lack of homogeneity among items [8]. In the present study, Cronbach’s alpha values for all of the IBS-HR-QOL total and subscales satisfied the criterion of 0.75–0.95, thus exhibiting excellent internal consistency reliability.

Test–retest reliability establishes the presence of temporal stability by repeated measurement in the same subjects [4]. The ICC and Pearson’s correlation coefficient are commonly used as parameters for measuring test–retest reliability. However, Pearson’s correlation coefficient does not take into account systematic differences between repeated measures, and the ICC is thus recommended as a more appropriate parameter [36]. The ICC was therefore used in the present study, and demonstrated satisfactory temporal stability of the IBS-HR-QOL over a 1-week period. Similarly, 1- or 2-week intervals were used in other studies involving the IBS-QOL [9, 23, 39, 40] and IBS-36 [11], in which their temporal stability was also satisfied. It may therefore be assumed that the attributes of HRQOL in IBS patients are stable for 1–2 weeks.

The IBS-HR-QOL developed in this study exhibited excellent psychometric properties. This instrument has practical strengths. First, there were few missing values for the IBS-HR-QOL items in the present study, which implies that this instrument is comprehensible to IBS patients. Second, this instrument, which comprises a total of 16 items, is shorter than other instruments: the IBSQOL, IBS-QOL, IBS-HRQOL, IBS-36, and IBS-IS comprise 30, 34, 26, 36, and 26 items, respectively. It can be assumed, therefore, that the IBS-HR-QOL may represent a lesser burden for patients, rendering it more feasible for use in clinical practice and research than these other instruments. However, there are some limitations when applying the IBS-HR-QOL. The first limitation is that no test of responsiveness was conducted to determine the ability of the IBS-HR-QOL to detect important clinical changes over time [27]. A longitudinal study is thus recommended to measuring the changes in HRQOL between before and after a therapeutic intervention for IBS patients. The second limitation is that the IBS-HR-QOL has been developed using a paper-and-pencil mode of administration. In a busy clinical practice, this mode of application can be a burden for health professionals in terms of requiring the dissemination, collection, and calculation of paper-based answers [41]. As an alternative, it is recommended for a future study to transform the present IBS-HR-QOL into a computer-mode instrument (e.g., utilizing a laptop or handheld computer).

## Conclusion

The IBS-HR-QOL comprises 4 subscales (bowel function, emotions, concerns about social activity, and consideration of foods) with a total of 16 items. The IBS-HR-QOL demonstrated good psychometric properties: content validity, factorial validity, criterion validity, known-groups validity, convergent validity, internal consistency reliability, and test–retest validity. The IBS-HR-QOL is easily comprehensible to patients, and shorter than similar instruments. It is therefore feasible for use in clinical practice and research. Further studies are needed to determine the responsiveness of the IBS-HR-QOL.

## Additional file

Additional file 1: IBS-HR-QOL.

## Abbreviations

IBS: Irritable bowel syndrome
HRQOL: Health-related quality of life
IBSQOL: Irritable bowel syndrome quality of life
IBS-QOL: Irritable bowel syndrome-quality of life
IBS-HRQOL: Irritable bowel syndrome health related quality of life
IBS-IS: Irritable bowel syndrome impact scale
EFA: Exploratory factor analysis
CFA: Confirmatory factor analysis
IBS-HR-QOL: Irritable bowel syndrome-specific health-related quality of life instrument
CVR: Content validity ratio
SF-36: Short form-36
IBS-SSS: Irritable bowel syndrome symptom-severity scale
HADS: Hospital anxiety and depression scale
KMO: Kaiser-Mayer-Olkin measure of sampling adequacy
GFI: Goodness-of-fit index
SRMR: Standardized root mean square residual
RMSEA: Root-mean-square error of approximation
CFI: Comparative fit index
NFI: Normed fit index
MGCFA: Multigroup confirmatory factor analysis
ICC: Intraclass correlation coefficient
MI: Modification indice
IFI: Incremental-fit index
