# Supplementary material for: Irritable bowel syndrome-specific health-related quality of life instrument: development and psychometric evaluation
Source: Health Qual Life Outcomes. 2016 Feb 17;14:22. doi: 10.1186/s12955-016-0423-9 (PMC4756460; doi:10.1186/s12955-016-0423-9)
Supplement: Additional file 1: — IBS-HR-QOL. [file 12955_2016_423_MOESM1_ESM.pdf]

Additional file 1.

과민성 장증후군 특이형 삶의 질

아래의 문항은 과민성 장증후군이 귀하의 삶에 어떤 영향을 미쳤는지에 대한 것입니다. 각 문항에 대해 **지난 한달 동안** 귀하의 상태가 어떠했는지 오른쪽 알맞은 칸의 **번호에 동그라미 표시**를 해 주십시오.

	문 항	전혀 그렇지 않았다	약간 그랬다	보통으로 그랬다	꽤 그랬다	많이 그랬다
1	과민성 장증후군으로 배가 빵빵하고 팽창된 느낌이 들었다.	0	1	2	3	4
2	과민성 장증후군으로 대변은 나오지 않으면서 아랫배만 묵직한 증상이 있었다.	0	1	2	3	4
3	과민성 장증후군 때문에 대변이 평소보다 딱딱해지거나 혹은 묽어졌다.	0	1	2	3	4
4	과민성 장증후군 때문에 대변보는 횟수가 평소보다 많아졌거나 줄어들었다.	0	1	2	3	4
5	과민성 장증후군으로 배에 가스가 차서 부글거렸다.	0	1	2	3	4
6	과민성 장증후군 때문에 우울했다.	0	1	2	3	4
7	과민성 장증후군으로 짜증이 났다.	0	1	2	3	4
8	과민성 장증후군으로 인해 다른 사람들한테 화를 냈다.	0	1	2	3	4
9	과민성 장증후군 때문에 신경이 예민해졌다.	0	1	2	3	4
10	과민성 장증후군 때문에 어디를 가도 화장실 위치를 먼저 확인해야 하는 번거로움이 있었다.	0	1	2	3	4
11	과민성 장증후군으로 인한 설사를 참지 못하고 공공장소에서 실수할까봐 걱정스러웠다.	0	1	2	3	4
12	과민성 장증후군이 언제 갑자기 나타날지 예측할 수 없어 불안했다.	0	1	2	3	4

13	과민성 장증후군의 증상이 나타날까봐 외출 전에 음식을 먹거나 마시지 않았다.	0	1	2	3	4
14	과민성 장증후군 때문에 먹는 음식에 항상 신경을 써야 했다.	0	1	2	3	4
15	과민성 장증후군 때문에 평소 좋아하는 음식을 먹을 수 없었다.	0	1	2	3	4
16	과민성 장증후군 때문에 외식하는 것이나 회식하는 것이 꺼려졌다.	0	1	2	3	4

▶**승인 없이 수정 및 사용 할 수 없습니다.**
